# High Rank, Low Tolerance: Hierarchy-Dependent Reactions of Cohabiting Companion Dogs to Being Separated from Their Owner

**DOI:** 10.3390/ani16131965

**Published:** 2026-06-25

**Authors:** Petra Dobos, Kata Vékony, Viktória Bakos, Blanka Veres, Csenge Anna Lugosi, Péter Pongrácz

**Affiliations:** 1 Department of Ethology, ELTE Eötvös Loránd University, 1117 Budapest, Hungary; dobpet2001@student.elte.hu (P.D.); kata.vekony.kami@gmail.com (K.V.); bakosvikee@gmail.com (V.B.); lugosipolly@gmail.com (C.A.L.); 2 Institute of Cognitive Neuroscience and Psychology, HUN-REN Research Centre for Natural Sciences, Magyar Tudósok krt. 2, 1117 Budapest, Hungary

**Keywords:** agonistic rank, formal rank, leadership rank, hierarchy, separation test, rank score

## Abstract

Cohabiting companion dogs establish hierarchy among themselves. It is hypothesized that the owner represents the main and undividable resource, thus primary access to this is a main organizing factor of rank-related behaviors of dogs. Here we tested high- and low-ranking cohabiting companion dogs’ (N = 70) reactions to their owner’s absence in a 3 min separation test. Rank scores have been assigned with a validated questionnaire (DRA-Q). We predicted that dominant dogs would show stronger reactions to being separated from their owner. Indeed, we found that higher-ranking dogs showed more intense activity and sooner arising attempts to leave the room (rearing at the wall, scratching the door, moving around, barking) than lower-ranking dogs did. These reactions may show also the intention to reestablish their connection with the absent owner. The associations between dogs’ rank and the behavioral responses were modified by the dogs’ age (negatively), the number of cohabiting dogs (positively), and we found that subcategories of the dog’s dominant status (such as ‘agonistic’ and ‘leadership’ subscales) were also associated with finer details of the outcome. These are the first results indicating that presence of the owner may provide more reassurance to higher-ranking dogs against stress than it does to lower-ranking dogs.

## Simple Summary

Although they are provided with all the necessary resources by their owners, there is still a well-established hierarchy among cohabiting companion dogs. Hierarchy affects not only resource-related behaviors, but also other social encounters, such as problem solving and social learning. We hypothesized that the owner represents a main and undividable resource for dogs and higher-ranking dogs would show more intense reactions as compared to the subordinate ones when they are being separated from the owner. In this study, we tested cohabiting dogs with known rank scores. One by one, dogs were left alone in a room, and we observed their reactions to the absence of their owner. We found that dogs with higher rank scores tried to get out of the room sooner and more vigorously: they reared at and scratched the wall more, barked sooner and they were also more active during the separation. These results indicated that higher-ranking dogs cope less easily with being separated from their owner, because they probably experience this situation as a threat to their primary access to their main ‘resource’ in the household.

## 1. Introduction

The dog–human relationship relies on a dependence-based coexistence [1,2]. Dog–human coevolution not only led to dogs that secure the necessary resources for themselves via amicable behaviors towards humans [3] but have also driven the adaptation of dogs towards such socio-cognitive capacities that maintain and nurture this connection with humans [4,5]. Among the wide range of these traits, we can mention the ability to learn from even unfamiliar humans [6]; multimodal and bi-directional communication (e.g., visual communication [7]; and acoustic modality [8]); sensitivity to ostensive communication (i.e., words and gestures produced in an attention-eliciting and -directing manner) [9] and empathy [10]. In the case of companion/working dogs, the owner has a central importance for dogs, which is signified by behavioral traits that are aimed to secure exclusivity for the dog to the owner (e.g., individual recognition of the owner’s face [11]; owner’s voice [12]; attachment [13]; referential communication [14]; and asking for help [15]. The importance of the exclusive bonds with the owner can be clearly detected when the dog’s relationship/access to the owner is threatened by social (e.g., jealousy’ against a social rival [16]) or environmental factors (e.g., physical separation from the owner [17]). When they experience this kind of situation, dogs show various forms of stress, and they may actively attempt to improve the situation.

Being separated from the owner is one of the main sources of stress for companion/working dogs [18]. The reaction of dogs to reestablish their connection with the owner can be regarded as biologically relevant and adaptive behavior. The fundamental source of separation-related stress in dogs is their attachment towards their owner which is similar to the parent–infant bond [19]. Separation disrupts the safety that was provided by the presence of the attachment figure (i.e., the owner) and consequently activates stress-response systems. When separation-related reactions exceed certain levels of severity, they are considered as problematic behavior, or, in particularly extreme cases, as being a behavioral problem [20]. Separation-related problematic behaviors manifest in vocalization, destructive behavior, indoor elimination and escape attempts [17,21].

Separation-related problems are very common in the companion dog population, and researchers put a considerable amount of effort into understanding their causation and provide potential solutions to amend the situation [22]. Among others, it was found that sex of the dog [23]; breed type (i.e., cooperative vs. independent working dog breeds [24]), and early-life upbringing factors [25] can all influence the occurrence of separation-related signs. It is known that the relationship between the dog and its owner [26,27], more specifically, the owner’s habits (e.g., being lenient or strict with the dog [28]) and behavior (e.g., [29]) can strongly affect the dog’s reaction to the separation. All in all, it seems plausible that any factor that can relate to the strength and quality of the dog–owner social bond, may also influence the dog’s responses to being separated from the owner. Here, we propose another ecologically valid approach to a dogs’ reaction to separation: the effect of between-dog social dynamics, in other words, hierarchy.

Hierarchy among cohabiting dogs [30] just as in other animals, reduces conflict in the distribution of resources [31]. From an ethological perspective, dominance can be understood as a qualitative measure based on the consistent outcomes of interactions over a resource [32]. Both formal (i.e., submission-based) and agonistic (i.e., aggression-based) dominance have been described in wolf packs [33,34]; however, aggressive behaviors are relatively rare among wild-living wolves [33]. Dogs and wolves share a common ancestor [35]; however, the domestication process of dogs resulted in different social organizations, driven by new, mostly anthropogenic ecological pressures. While in nature, the hierarchy within wolf-packs usually develops as a natural, age-related consequence between parents and their offspring [36], in the case of dogs, cohabitance is very rarely connected to kin-related connections. Among dogs, the number of cohabiting animals widely varies between the commonly occurring single-kept companion dogs to the larger, ad hoc (e.g., in dog parks [37]) or more-or-less permanent groups [38]. Free-ranging dogs (FRDs), unlike wolves [39], are usually only distantly related with other members of the group [40] and cohabiting companion dogs usually come from various (non-related) sources [41]. Although there are indications that show alloparental behavior may occur among the cohabiting animals in dog breeders’ homes, where sometimes the puppies’ father or other, closely related females occur [42], there is no predictable, standard biological kinship structure in human-governed dog groups. To our best knowledge, there were no studies that systematically investigated the ratio of relatedness among cohabiting dogs.

Despite the lack of kinship-based and limited resource-based organizing factors that are typical in wild-living dogs (e.g., dingoes [43,44]; and free-ranging dogs [45]), hierarchy structures have also been described among companion dogs. These can develop among temporary aggregations of dogs in a daycare [46]; however, we have the most instances of rank-related behavioral differences from cohabiting companion dogs [47,48,49,50]. Dominance relationships of companion dogs are routinely assessed via questionnaire methods, where the owner provides information about the behaviors that may indicate the higher or lower status of a dog in the social system at home [49,51]. The validity of these questionnaire methods was also confirmed with behavioral observations [51,52]. It is also important to see that beyond using a ‘holistic’ rank score for the cohabiting dogs that tells whether an individual is either above or below the other, dominance can be further categorized according to three behavioral displays: agonistic rank, formal rank and leadership rank [52]. Regarding agonistic rank, in competitive scenarios higher-ranking individuals exhibit assertive behaviors and lower-ranking individuals show consent and submissive behaviors. Formal rank covers behaviors (e.g., licking the higher-ranking partner’s mouth) that are related to the rank of the individual besides competitive situations and reinforces hierarchy without fighting. Leadership rank consists of initiative behaviors such as barking earlier or more at strangers, walking in front and defending the group. These three types of behaviors can vary among individuals and contexts, where the higher-ranking dog may not maintain dominance in every scenario [51]. With using a more detailed approach to rank-related behaviors in dogs, we can reach a more flexible and detail-oriented assessment of the various contexts where between-dog social dynamics can play a role.

Beyond the usual commodities that may elicit competition (e.g., food, toy, reproductive partner, etc.), the most important (and undividable) resource for dogs is the owner [53]. The owner has a crucial role in the lives and success of dogs, due to the possession and distribution of the resources. As dogs with an owner normally do not need to or are not allowed to compete for food, toys, resting places or sexual partner, they will compete for the attention of the owner, who provides these resources. There are also empirical indications that low- and high-ranking dogs relate differently to their owner. Subordinate dogs were found to learn a detour task successfully from their cohabitant dominant companion, but they could not improve in their success rate if the owner showed the action [50]. Moreover, in an attachment test, dogs with higher rank stressed less in the owners’ presence and displayed fewer friendly behaviors towards the stranger during the test [54]. These studies suggest that while dominant dogs interact and rely more on their owner, subordinate dogs are more dependent on the higher-ranking conspecific, who gains more access to the owner and leads the cohabiting dog group.

If the owner represents a fundamental social resource for companion dogs, safely securing it from the competitors may also include being uninterruptedly in the vicinity of the owner. In our present study, we investigated how cohabiting companion dogs would react to the absence of their owner. To the best of our knowledge, this is the first study where the association of dogs’ ranks on their reactions to separation from their owner was analyzed. We examined separation-related behaviors in a scenario where each dog was left alone in an unfamiliar room for 3 min. We hypothesized that dominant and subordinate dogs would react differently to the absence of their owner, according to the already established order of access to this ‘omnipotent resource’. We predict that dominant dogs, who have already secured primary access to the owner, will show more signs of frustration when the owner is absent than the subordinate dogs would exhibit (e.g., trying more adamantly to leave the room; barking; walking around, etc.). However, an alternative prediction could be that dominant dogs would react with less fear/higher confidence to separation, as they have a safer, more stable position in the hierarchy at home (e.g., less whining; less panting; more activity, etc.).

## 2. Materials and Methods

### 2.1. Subjects

We tested N  =  70 adult (1 year or older: M_age_ = 6.76 ± 3.31 years) companion dogs (purebreds and mixed breeds) from N = 32 multi-dog households. Most participating dogs were spayed/neutered (37 females and 25 males), and there were only 4 intact males and 4 intact females among the subjects. The basic demographic details of the subjects are provided in Supplementary Table S1. At least two, and a maximum of three subjects were included in the study from each household. Dog owners were recruited through advertisements placed on social media and the dogs were tested in the presence of their owner. Participation was voluntary, and each owner signed an informed consent form. The holistic rank scores and three subscores (agonistic, formal, and leadership) of the participating dogs were determined by a validated questionnaire (DRA-Q, [53]) completed by the dog owners.

### 2.2. General Procedure

All tests took place in the laboratory (6.27 × 5.4 m testing room) of the ELTE Department of Ethology, Budapest, Hungary. We ran all tests between November 2024 and February 2025. Subjects were tested only once. After arriving at the testing site, dog owners gave their written informed consent; they entered the department with the researcher, who was always a young woman (C.A.L. or B.V.). The E explained to the owners how to behave during the test. We followed the methodology of Konok et al. [27]. During this test, the room was empty except for a chair. The owner entered the room with the dog on leash. After taking off the leash the dog was free to explore the room during the entire test. At first the owner sat on the chair and did not initiate any interaction with the dog. After 1 min elapsed (measured with a stopwatch by the owner), the owner left the room without any interaction with the dog, leaving the dog’s leash on the chair. The dog was alone in the room for 3 min. After the 3 min elapsed, the owner returned, greeted and pet the dog. For behavior coding, we used only the interval that started when the owner closed the door after leaving the room and finished when the owner opened the door again. In both tests, the dog’s behavior was recorded from above with a digital camera system and two microphones (Basler a2A1920-51gcPRO-Basler ace 2, Basler AG, Ahrensburg, Germany; microphone: Sennheiser ME-64 + K6-P power module, Sennheiser Electronic GmbH & Co., Wedemark, Germany; and audio interface: Focusrite-Scarlett 2i2, Focusrite PLC, Wycombe, UK). Later we used these recordings for data extraction.

### 2.3. Sample Size Calculation

We determined the desired sample size by using the equation for finite populations:

(1) $$ń=\frac{n}{1+\frac{z^{2}\;\times \;\hat{p}(1\;-\;\hat{p})}{\varepsilon^{2}N}}$$

where z (z-score) = 1.96 for the 95% confidence level, ε (margin of error) = 0.05, and $\hat{p}$ (population proportion) = 0.50. We expected that the population of suitable dogs (N) for our test would be 60 (based on previous social media subject recruiting campaigns and a reasonable timeframe, we could invite no more than 60 subjects from multi-dog households). The calculated sample size was N = 53. We had the opportunity to test a slightly higher number of subjects (N = 70), expecting that some of the subjects would need to be excluded for various reasons (technical issues, and non-complying owner). At the end, we could use data from each subject in the statistical analysis.

### 2.4. Behavioral Coding

Each test was video recorded. We used Solomon Coder (beta 19.08.02, Copyright by András Péter) for the extraction of data from the video sequences. Table 1 shows the behavioral variables we used for the analysis. For reliability testing, 11% of the dogs were coded by an independent coder.

### 2.5. Statistical Analysis

We used R statistical software (version 4.4.1, R Core Team, Vienna, Austria, 2024) in RStudio (2026.01.0 +392, RStudio Team, Boston, MA, USA) with packages AICcmodavg, corrplot, coxme, DataExplorer, emmeans, fitdistrplus, glmmTMB, GPArotation, irr, outliers, paran, performance, psych and rstatix.

We calculated the factor scores according to the component structure originating from the Principal Component Analysis (PCA) based on Lenkei et al. [55], whose behavioral scoring method we also used in the present study. The five components (behavioral dimensions) accounted for 56.3% of the total variance. (The detailed structure of the principal components is shown in Supplementary Table S2).

We calculated the ICC (Inter Class Correlation) values on the coded behaviors to ensure inter-rater reliability. In the case of latencies of individual behaviors, we found moderate to excellent consistency between the two coders (rearing ICC = 0.652; scratching ICC = 1.000; barking/yelping ICC = 0.912; whining ICC = 0.787; other vocalization ICC = 1.000). In the case of the five components resulting from the PCA, the two coders had moderate to excellent consistency (chair ICC = 0.852; escape ICC = 0.760; Bark–wagging ICC = 0.748; Sit ICC = 0.994). We retained these components for further analysis. In the case of the fifth component (whine–door) the between-coder consistency was poor (ICC = 0.356); therefore, we removed this component from the main analysis.

We used the resulting PC factor scores as dependent variables in further analyses. We used Generalized Linear Mixed Models with the household ID as a random effect and rank scores from the DRA-Q and demographic data as predictors. We also used Mixed Effects Cox Regression Models to analyze the association between DRA-Q scores and the latency to perform certain specific behaviors (barking, whining and overall vocalization; and behaviors related to escape attempts: scratching and rearing). Overall vocalization was calculated by combining all measured vocalization types. For latency of ‘overall vocalization’, we used the latency of the very first vocalization regardless of type (barking, whining or ‘other’). For durations, we added together the durations of all vocalization types. We used AIC-based model selection using the overall rank score and the subscores separately, then we compared the final models to find the best fit.

## 3. Results

**Figure 1:**
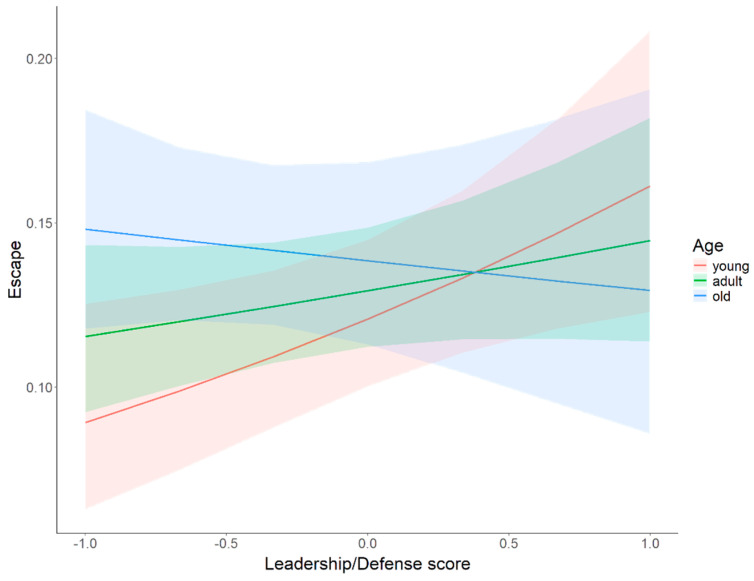
Association between the escape principal component score and the interaction between leadership/defense DRA-Q subscore and age of the dogs. Dogs scoring higher on leadership/defense, tried to escape more, but this effect became weaker with age. Age was used as a continuous variable, categorized for visualization purposes only. The three age categories read as the following: young = [MinimumAge: MeanAge − SDAge]; adult = (MeanAge − SDAge: MeanAge + SDAge); old = [MeanAge + SDAge − MaxAge].

You can see the descriptive statistics of the main behavioral parameters and the behavioral dimensions from the PCA in Supplementary Table S3. The main association revealed by the GLMMs was between the escape principal component and rank. In the models using the holistic rank score, there was a significant interaction effect with the number of cohabiting dogs. According to the post hoc test, dogs with higher rank scores showed stronger escape if they came from households where there were more than two dogs (β = 0.787, SE = 0.374, z = 2.102, 95%CI = (0.0533–1.5202), *p* = 0.0355). Using the subscores in the model revealed a significant association between the leadership/defense score and the ‘escape’ PC (β = 0.550, SE = 0.227, z = 2.423, 95%CI = (0.1052–0.9951), *p* = 0.0154), and also a significant interaction between leadership/defense and age (β = −0.062, SE = 0.031, z = −2.034, 95%CI = (−0.1219–0.0023), *p* = 0.0419, Figure 1): dogs that scored higher on the leadership/defense scale tried to escape the room more, but this effect decreased with age. This model fits better than the one with the rank score (∆AIC = 3.42). Additionally, we checked if dogs’ age correlated with the leadership/defense subscore, where we found a significant negative effect (R_s_ = −0.3350, t = −2.932, 95%CI = (−0.5284, −0.1086), *p* = 0.0046).

**Figure 2:**
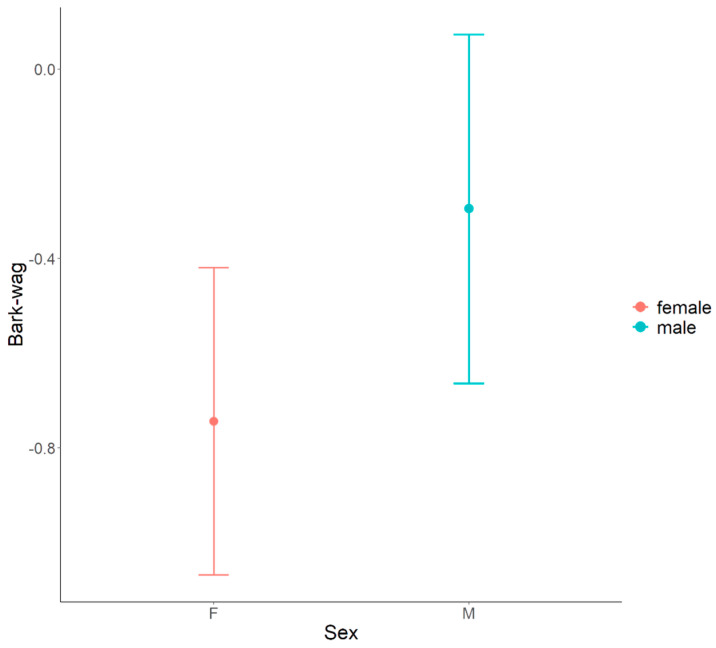
Association between the Bark–wagging principal component score and sex. The figure shows the means and confidence intervals. Male dogs barked more during the test.

The component ‘Bark–wagging’ showed significant association with dogs’ sex: male dogs barked and wagged their tail more while they were orienting to the door (β = 0.486, SE = 0.203, z = 2.392, 95%CI = (0.4273–0.9756), *p* = 0.0197, Figure 2). The components ‘chair’ and ‘Sit’ did not show associations with any of the fixed factors.

**Figure 3:**
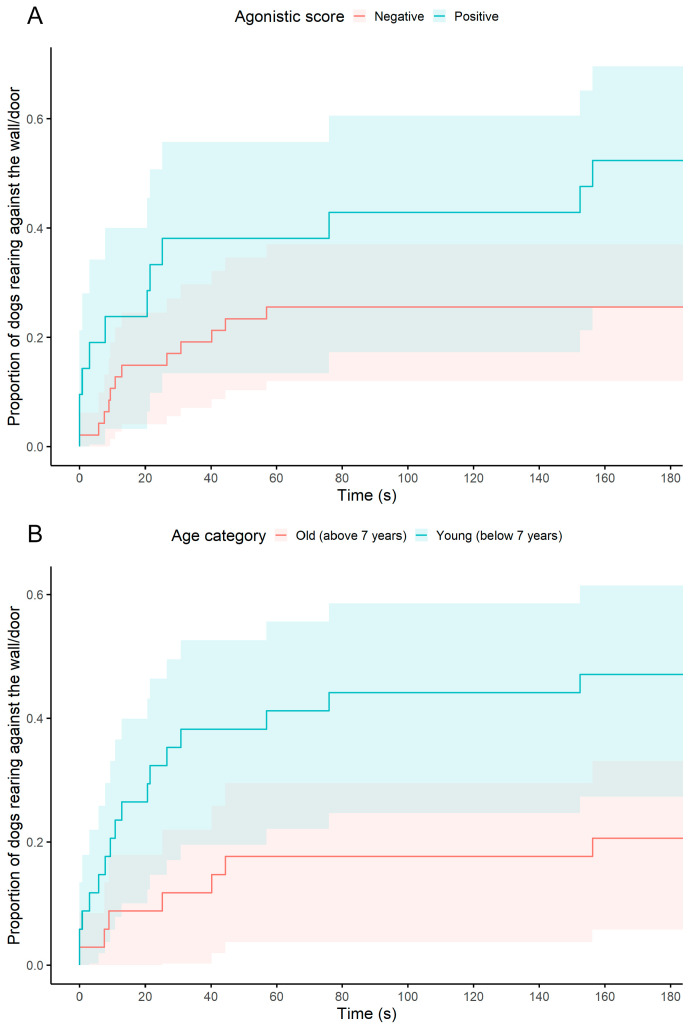
The associations between agonistic hierarchy scores (**A**) and dogs’ age (**B**) with latency to start rearing against the door or the walls. Dogs scoring higher on the agonistic subscale of the DRA-Q were more likely to rear, and started sooner (**A**), as well as the younger dogs (**B**). Both age and agonistic score were measured as continuous, and here we only categorized them for visualization.

**Figure 4:**
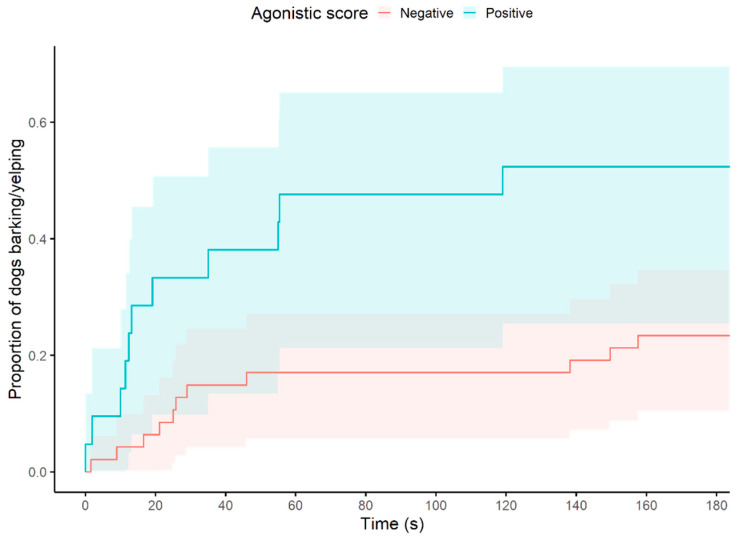
Association between the latency to start barking and the agonistic subscale of the DRA-Q. Dogs scoring higher were more likely to bark and started barking sooner than lower scoring dogs.

When analyzing the latencies of individual behavioral elements, rank score and dogs’ age had a near-significant trend-like effect on the latency of rearing (rank score: exp(β) = 3.480, SE = 0.669, z = 1.86, 95%CI = (−0.0637–2.5579), *p* = 0.0622; age: exp(β) = 0.863, SE = 0.079, z = −1.86, 95%CI = (−0.3016–0.0081), *p* = 0.0632). Dogs’ age also had a non-significant trend with barking (exp(β) = 0.879, SE = 0.075, z = −1.72, 95%CI = (−0.2750–0.0175), *p* = 0.0846). These results seem to indicate that higher-ranking and younger dogs may start to rear at the door and wall somewhat sooner and younger dogs may start to bark sooner; however, we would need further research to verify this on a larger sample. Analyzing the subscores revealed a significant association between the latency of rearing and the agonistic score and age: higher scoring and younger dogs started to rear sooner, respectively (agonistic score: exp(β) = 3.486, SE = 0.540, z = 2.31, 95%CI = (0.1896–2.3079), *p* = 0.0208; age: exp(β) = 0.846, SE = 0.081, z = −2.05, 95%CI = (−0.3271–0.0075), *p* = 0.0402, Figure 3). This model had a better fit than the one using rank score (∆AICc = 2.28). In the case of barking, we found that agonistic score had a significant effect: higher scoring dogs started to bark sooner (exp(β) = 2.778, SE = 0.505, z = 2.02, 95%CI = (0.0324–2.0107), *p* = 0.0429, Figure 4). The model with the agonistic score and the one with age had similar fits (∆AICc = 1.69). We did not find any association of these fixed factors with whining, other vocalizations, summarized vocalizations and scratching.

## 4. Discussion

The main goal of our study was to investigate whether there were associations between the companion dog’s rank in their established hierarchy at home and their separation-related behaviors. According to our hypothesis, among cohabiting dogs, the temporary absence of the owner (i.e., the main ‘social resource’) would cause rank-dependent differences in their reactions to separation.

We found that higher-ranked dogs reared at the door more often and sooner, and tried to get out of the room more actively than the lower-ranked dogs did. The assumed higher motivation to reestablish contact with their owner is in line with our predictions and previous findings. Vékony et al. [53] showed association between personality traits and social rank where the more ‘conscientious’ dogs tended to rank higher. In the Canine Big Five instrument, the trait ‘Conscientiousness’ includes items related to being focused, persistent, and goal-oriented [53] and these traits are also important in sport or work engagement of the dogs. Probably higher-ranked dogs that are more enthusiastic during dog–human activities, also prefer active escape attempts when they are left alone. Vékony et al. [56] tested cohabiting low- and high-ranking dogs in a scenario where the dogs faced a frustration-eliciting situation: namely, when a previously accessible treat became inaccessible either because the researcher withheld it (social test), or it was in a closed cage (non-social test). In general, dominant dogs showed more behaviors that indicated their owner-dependency in the non-social test than in the social one, while subordinate dogs’ owner-directed behaviors did not change. In the same study, subordinate dogs tried to obtain the unreachable food on their own, while higher-ranking dogs sought the help of their owner [56]. Subordinate dogs also showed fewer human-dependent behaviors, leading to consequently weaker performance in social learning tasks, where the task was demonstrated for them by an unfamiliar experimenter [47] or by their owner [50]. Finally, in the Strange Situation Test, which was designed to assess dogs’ attachment towards their owner, higher-ranking dogs showed less signs of stress and lower sociability towards a friendly stranger when their owner was also present [54]. According to the authors, dominant dogs may rely more on their owner than subordinate dogs do, and the presence of the owner provides more reassurance to the higher-ranking dogs against stress than it does to the lower-ranking dogs. One could also argue that active escape-related behaviors can more easily emerge in a situation when the owner is not present, because there is no one that would intervene with calming or discipline of the dog. This factor, however, would be true for both the lower- and higher-ranking dogs; thus, it in itself could not explain the rank-related differences we found here.

We showed that higher-ranking dogs from households with three or more dogs tried to escape more actively than those who lived with only one other conspecific. Earlier theoretical considerations in the case of primates in general [57] and empirical results in Rhesus macaques [58] showed that resource competition increased with larger group sizes. Our results suggest that living with more competing individuals can positively correlate with higher-ranked dogs’ dependency on the owner, which could be the consequence of the more intense competition for the main ‘resource’. Within-group resource competition is expected to be lower in highly cooperative groups (as in the case of gray wolves [25]; or African wild dogs [59]. However, in the case of dogs, the willingness to cooperate with conspecifics was found to be less pronounced than in wolves [60]; thus, our results in which we found stronger effects of social isolation from the owner on higher-ranking dogs living with more conspecifics, fit well to this low-cooperativity/stronger competition model.

We also found significant association between leadership/defense subscores, dog’s age and active escape attempts. Younger dogs with higher leadership/defense subscores tried to escape from the room more actively, but older dogs with higher leadership/defense subscores did not show the same tendency. This result is in good alignment with the associations between dogs’ ranks and their Canine Big 5 personality scores found by Vékony et al. [53]. In that study, rank score showed a positive association with ‘Extroversion’, a trait that includes behavioral items favoring active solutions. At the same time, dogs’ age was in a negative association with their extroversion; thus, one can expect that older dogs, even with higher rank scores, would show decreasing levels of active attempts to get out of an unpleasant situation. In accordance with this assumption, we also found that older dogs barked less and spent less time rearing on the door than younger ones. Barking can be a sign of frustration and anxiety in the case of a separation situation [61]. Bakos and colleagues [54] found that older dogs showed less signs of stress during the Strange Situation Test, which also incorporates short periods of separation from the owner. Our results are in line with previous findings of an age-related decrease in social attention [62] (e.g., frequency of looking at the owner, duration of eye contact). We also should take into consideration that leadership/defense subscore showed a negative correlation with dogs’ age, which means that the young dominant dogs shown more intense dependency on humans. An alternative explanation can be that younger dogs usually have higher activity levels than older ones; therefore, they prefer active escape attempts. It is also conceivable that older dogs have already encountered the absence of the owner many times, which is why they now show less signs of agitation in such situations.

Interestingly, dogs with higher agonistic subscores showed rearing on the door more and sooner and barked more than dogs with lower agonistic subscores. While escape attempts include more active locomotion, which is seemingly connected to higher-ranked dogs with higher lead/defense subscores, rearing on the door is a more goal-oriented behavior probably shown by dogs with higher agonistic subscores, who prefer the more direct and effective solutions [50,51].

Furthermore, males barked more and showed more tail-wagging than females, which fell in line with previous results. Sargisson et al. [63] and Bradshaw et al. [23] also found that male dogs were more likely to show separation-related behaviors. Other studies found no association between dogs’ sex and separation-related behavior [17,64]. It is also conceivable that our sample accidentally included more males prone to separation anxiety than females.

Non-frustration-related behaviors (e.g., fear) were not associated with dogs’ rank; moreover, we did not find an association between passive behaviors (such as sitting or interacting with the chair) and other confounders. This result indirectly supports the assumption that a dogs’ rank is in connection with such reactions in a separation situation, which could be connected to frustration-related, and in general, active attempts as a response to the encountered problem.

Here, we should emphasize that throughout our research, we considered dogs’ rank score as a relatively stable attribute of the cohabiting dogs at the time of the testing. There was a minimum boundary of 6 months that the dogs had to be with the owner, but other than that, we did not test the possible effects of the length of time the dog spent with its owner and the other dog(s). Although the position of dogs in the hierarchy is secured through dynamic interactions, and the ‘holistic’ rank consists of various components (‘agonistic’, ‘formal’, and ‘leadership’), the multi-question DRA-Q instrument provides the opportunity for reliable and biologically relevant assessment of the structure of an established rank system among cohabiting dogs [56]. While environmental and demographic factors may influence the changes in the individual dog’s rank, in our investigation, we tested adult subjects who have already spent a long enough time in mutual cohabitation for the development of stable rank conditions. The complex interrelationship between dogs’ rank and their personality traits [53] could also provide relative stability to dogs’ position in the hierarchy, protecting it from temporary fluctuations in strength and motivation, caused by, for example, health conditions or environmental stress.

As separation-related behaviors can lead to the relinquishment of dogs to a shelter [18], it is important to identify potential predisposing factors that owners can take into account when trying to understand their dogs’ behavior. Our results are important in terms of animal welfare and successful dog–human coexistence, because we show for the first time that dogs’ rank could affect separation-related behaviors of dogs.

## 5. Conclusions

In our present study, we investigated how cohabiting companion dogs would react to the absence of their owner. If the owner represents a fundamental social resource for companion dogs, safely securing it from the competitors may also include being uninterruptedly in the vicinity of the owner. Our results support this approach, as dominant dogs (especially younger ones), who have already secured primary access to the owner, showed more signs of frustration when the owner was absent, and tried to get out of the room more actively and reestablish contact with their owner. Dominant dogs may rely more on their most important resource (the owner) than subordinate dogs; thus, presence of the owner provides more reassurance to the higher-ranking dogs against stress than it does to lower-ranking dogs.

We also found that higher-ranked dogs from households with three or more dogs tried to escape more actively than those that lived only with one conspecific, suggesting that living with more competing individuals is positively correlated with higher-ranked dogs’ dependency on the owner.

While in other studies the rank subscores had divergent associations with the dogs’ behavioral responses in a given task or situation, and their relevance was strongly context-dependent [51,56]; here, they also pointed in the same direction. This suggests that separation from the owner might be one of the few contexts where all aspects of rank have similar relevance. The agonistic score is an aspect of rank that is based completely on resource-related behaviors [45] which affected their determined, independent, persistent attempts to reach the resource (here, the owner). Leadership score is an aspect of rank that relies highly on social competence [48]. As companion dogs in multi-dog households have no more important ‘resource’ to compete for other than the owner’s attention, vicinity, and interactions [53], higher-ranked dogs with both higher leadership and agonistic scores, had the motivation to show more active escape attempts in the absence of the owner.

## 6. Limitations

We acknowledge some limitations of the study; for example, we could not investigate if the duration of dogs’ cohabitance would influence their behavior during the tests. Time spent together might affect social behavior directly, but this effect might be mediated through the hierarchy which also takes time to establish. Our subjects were adult dogs living together for at least 6 months, which could be enough to establish hierarchy, but we did not ask the owners about possible recent changes in their dog group, such as adopting a new dog or death of an older dog that can also induce changes in the hierarchical structure among the cohabiting dogs.

Separation-related behavior can be affected by several other factors that are difficult to control—early maternal separation [65], living in a single-owner household [17], and sudden changes in the life of the dog (e.g., moving, loss of the owner) can lead to increased separation-related problems [63]. Furthermore, we could not assess how much time dogs usually spend alone at home or in relatively strange places (e.g., during holidays), which could also influence our results.

## Figures and Tables

**Table 1:** The list and descriptions of behavioral variables that we used during video coding.

Behavioral Variable	Unit	Description
Stand	Duration	The dog is on four feet, not moving
Sit	Duration	The dog’s haunches are on the ground, but the elbows are not
Lay down	Duration	The dog’s elbows and sternum or side touch the ground
Move	Duration	The dog is moving, walking, or running, 2–3 paws are on the ground the whole time
Chair distance	Duration	The dog is near to the chair, within one body length
Door distance	Duration	The dog is near to the door, within one body length
Rearing	Duration and latency	The dog stands on his/her hind feet and puts the forelegs on the doors or the walls
Scratching	Duration and latency	The dog scratches the doors or the walls, with his/her forelegs, or tries to open the door by pawing the handle
Orientation towards the door	Duration	The dog’s head is pointing towards the door
Orientation towards the chair	Duration	The dog’s head is pointing towards the chair
Exploration in general	Duration	Activity directed toward physical aspects of the environment, including sniffing, close visual inspection, distal visual inspection, and gentle oral examination such as licking
Chair exploration	Duration	Activity directed towards the chair, including sniffing, close visual inspection, distal visual inspection and gentle oral examination such as licking
Door exploration	Duration	Activity directed towards the door, including sniffing, close visual inspection, distal visual inspection, and gentle oral examination such as licking
Barking/yelping	Duration and latency	A loud, short, wide pitch and range sound with inverted U-shaped pitch contour
Whining	Duration and latency	High-pitched, relatively tonal, short and cyclic, or elongated vocalizations.
Panting	Duration	While the mouth is open, the noise made by the dog sounds like a loud, moderate to rapid, open-mouth respiration

## Data Availability

The dataset used for the analyses is provided as Supplementary Materials.

## Supplementary Materials

The following supporting information can be downloaded at https://www.mdpi.com/article/10.3390/ani16131965/s1. Table S1. Basic demographic details of dog subjects. The rank score values were calculated with the help of the questionnaire completed by the owners (originally published by Vékony et al., 2022 [53]). Dogs with same household numbers were cohabiting with each other. Table S2. The structure of the five behavioral components, resulting from the Principal Component Analysis. In this study, we used the identical scoring system of the behaviors in the separation test as Lenkei et al. (2021) [55] did, thus, we relied on the component structure they described as well. The table shows the factor loadings for each coded behaviors. Factor loadings with bold indicate which principal component the behavior belongs to. Table S3. Descriptive statistics of the Principal Components (behavioral dimensions) and individual behavioral variables we were using in the statistical analysis. We show the values separately for the dominant and subordinate dogs, where the within-household rank relationship was possible to establish with the help of the rank scores.
