# Supplementary material for: High Rank, Low Tolerance: Hierarchy-Dependent Reactions of Cohabiting Companion Dogs to Being Separated from Their Owner
Source: Animals (Basel). 2026 Jun 25;16(13):1965. doi: 10.3390/ani16131965 (PMC13359868; doi:10.3390/ani16131965)
Supplement: Supplementary file 1 [file animals-16-01965-s001.zip › Pongracz_Owner consent form.pdf]

Etológia Tanszék

Eötvös Loránd Tudományegyetem
1117 Budapest, Pázmány P. sétány 1/C
e-mail: etologiatanszek@ttk.elte.hu
http://etologia.elte.hu
☎ +36 1 381 2179

Declaration of Dog Owner's Consent

I, ...

E-mail (IN CAPITAL LETTERS):..

hereby confirm that I and my dog(s) participate in the ethological research conducted by the ELTE-MTA Családi Kutya Program voluntarily and at my own risk.

Dog's name		Breed	Sex	Age (years)

I have have been informed about the study and understand the aims and procedures of this research.[1]	**yes**	**no**
By participating in this research I give permission for my anonymized data: to be used in the context of this research and to be shared for the purposes of this research, in the form of statistically analysed results published in scientific and educational reports – in compliance with pertinent data protection laws.	**yes**	**no**
I understand that all of my personal or identifying information (names, e-mail addresses) will be stored separately from all other information, so that my personal and research data cannot be connected in any way.	**yes**	**no**
I am aware that I may discontinue participating in this research at any time (without penalty or loss of benefits to which I am otherwise entitled).	**yes**	**no**
I understand that this research will be videotaped for scientific purposes (only researchers will have access to/be able to view the videos).	**yes**	**no**
I approve of the videos being used for illustrative purposes (e.g., at conferences, in press releases).	**yes**	**no**
I confirm that, to the best of my knowledge, the above-named dog(s) is/are free from infectious diseases, has/have received all officially required vaccinations, and is/are not in heat.	**yes**	**no**
Should my dog(s) exhibit symptoms of an illness at any time throughout the duration of this study, I will take him/her/them to the veterinarian and inform the research staff about any illnesses or medical treatments and also about any changes in the animal's/animals' condition that may impact the results of the research.	**yes**	**no**
I declare that I keep my dog(s) leashed on campus (1117 Budapest, Pázmány Péter sétány 1/C) and take care that he/she/they does/do not jump on the ground floor parapet that conceals a ditch. I also declare that any relevant injuries or damages are my responsibility.	**yes**	**no**
Please, inform me when a summary of the research becomes available and on what website it is made available to the public.	**yes**	**no**

Date: Budapest, ...

Completed by the Researcher:
Name of study: ....

Researcher: ....

....

dog owner's signature

[1] Please, underline/circle your response to each of the statements below.
