# Supplementary material for: High Rank, Low Tolerance: Hierarchy-Dependent Reactions of Cohabiting Companion Dogs to Being Separated from Their Owner
Source: Animals (Basel). 2026 Jun 25;16(13):1965. doi: 10.3390/ani16131965 (PMC13359868; doi:10.3390/ani16131965)
Supplement: Supplementary file 1 [file animals-16-01965-s001.zip › Supplementary_Table3.pdf]

Supplementary Table S3. Descriptive statistics of the Principal Components (behavioral dimensions) and individual behavioral variables we were using in the statistical analysis. We show the values separately for the dominant and subordinate dogs, where the within-household rank relationship was possible to establish with the help of the rank scores.

Variable	measure	Dog's rank	Mean; SE	Min. – Max.
Chair (PC)	Factor score	Subordinate	0.90; 0.39	-2.87 – 6.80
		Dominant	0.40; 0.34	-2.55 – 5.03
Escape (PC)	Factor score	Subordinate	-0.18; 0.10	-1.66 – 1.09
		Dominant	0.04; 0.13	-1.01 – 1.92
Bark-wagging (PC)	Factor score	Subordinate	-0.66; 0.21	-2.39 – 1.85
		Dominant	-0.41; 0.17	-2.65 – 1.68
Sit (PC)	Factor score	Subordinate	0.06; 0.23	-1.44 – 2.85
		Dominant	0.22; 0.22	-1.01 – 3.25
Barking/yelping	Latency (s)	Subordinate	184.96; 17.73	1.60 – 240.00
		Dominant	154.78; 19.73	0.00 – 240.00
Whining	Latency (s)	Subordinate	69.14; 17.72	0.40 – 240.00
		Dominant	75.39; 18.17	1.40 – 240.00
Rearing	Latency (s)	Subordinate	185.14; 18.37	0.00 – 240.00
		Dominant	145.53; 19.87	0.00 – 240.00
Scratching	Latency (s)	Subordinate	224.98; 10.48	8.00 – 240.00
		Dominant	199.08; 15.44	0.00 – 240.00
Barking/yelping	Duration (s)	Subordinate	1.93; 0.97	0.00 – 19.00
		Dominant	4.21; 1.54	0.00 – 30.80
Whining	Duration (s)	Subordinate	26.54; 5.71	0.00 – 85.60
		Dominant	21.68; 4.90	0.00 – 117.80
Rearing	Duration (s)	Subordinate	2.66; 1.42	0.00 – 29.60
		Dominant	2.88; 1.72	0.00 – 51.40
Scratching	Duration (s)	Subordinate	0.46; 0.26	0.00 – 6.20
		Dominant	3.05; 2.68	0.00 – 80.40
