# Supplementary material for: High Rank, Low Tolerance: Hierarchy-Dependent Reactions of Cohabiting Companion Dogs to Being Separated from Their Owner
Source: Animals (Basel). 2026 Jun 25;16(13):1965. doi: 10.3390/ani16131965 (PMC13359868; doi:10.3390/ani16131965)
Supplement: Supplementary file 1 [file animals-16-01965-s001.zip › Table_Supplementary_1.pdf]

Supplementary Table S1. Basic demographic details of dog subjects. The rank score values were calculated with the help of the questionnaire completed by the owners (originally published by Vékony et al., 2022 [53]). Dogs with same household numbers were cohabiting with each other.

Household	Dog ID	Rank	Rank Score	Age (years)	Sex	Reproductive status
(1)	1	Subordinate	-0.125	5	female	spayed
(1)	2	Dominant	0.125	5	female	spayed
(2)	3	Dominant	0.125	4.2	male	neutered
(2)	4	Subordinate	-0.125	7	female	spayed
(3)	5	Subordinate	-0.25	2.5	male	neutered
(3)	6	Dominant	0.25	1.9	male	neutered
(4)	7	Subordinate	-0.75	2.5	female	spayed
(4)	8	Dominant	0.75	3.5	female	spayed
(5)	9	Subordinate	-0.375	10	female	spayed
(5)	10	Dominant	0.375	10	female	spayed
(6)	11	Subordinate	-0.5	10.5	female	intact
(6)	12	Dominant	0.5	5.5	female	spayed
(7)	13	Dominant	0.25	2	male	intact
(7)	14	Subordinate	-0.25	8	female	intact
(8)	15	Subordinate	-0.125	3	female	spayed
(8)	16	Dominant	0.125	3	male	neutered
(9)	17	Subordinate	-0.125	10	female	spayed
(9)	18	Dominant	0.125	12	male	neutered
(10)	19	Dominant	0	2	male	intact
(10)	20	Subordinate	-0.75	10	female	spayed
(11)	21	Subordinate	-0.375	5	female	spayed
(11)	22	Dominant	0.375	9	male	neutered
(12)	23	Dominant	-0.25	10.75	male	neutered
(12)	24	Subordinate	-0.5	12	male	neutered
(13)	25		0	7	female	spayed
(13)	26	Dominant	0.25	6	female	intact
(13)	27	Subordinate	-0.5	4	female	spayed
(14)	28		-0.25	8	female	spayed
(14)	29	Subordinate	-0.5	5	male	neutered
(14)	30	Dominant	0.25	7	female	spayed
(15)	31	Subordinate	-0.375	4	male	neutered
(15)	32	Dominant	0.375	5.5	female	spayed
(16)	33		-0.25	3	male	neutered
(16)	34		-0.25	7	female	spayed
(16)	35	Dominant	0	2.5	female	spayed
(17)	36	Subordinate	-0.375	11	male	neutered
(17)	37	Dominant	0.125	9.5	male	intact
(17)	38		-0.125	6	female	spayed

(18)	39	Subordinate	-0.375	5	male	neutered
(18)	40	Dominant	0.375	9.5	female	spayed
(19)	41		-0.125	8	female	spayed
(19)	42		-0.125	8	female	spayed
(20)	43	Dominant	0	11	male	neutered
(20)	44	Subordinate	-0.83333	5	male	neutered
(21)	45	Dominant	0.375	4.5	male	neutered
(21)	46	Subordinate	-0.375	3.5	female	spayed
(22)	47	Dominant	0.125	4.5	female	spayed
(22)	48	Subordinate	-0.125	4.5	female	spayed
(23)	49		-0.125	12.8	male	neutered
(23)	50	Dominant	0.125	5.5	female	spayed
(23)	51	Subordinate	-0.625	14	female	spayed
(24)	52	Fluid	0	2.5	male	neutered
(24)	53	Fluid	0	1.5	male	neutered
(25)	54	Subordinate	-0.375	8.5	male	neutered
(25)	55	Dominant	0.125	9.5	female	spayed
(26)	56	Subordinate	-0.5	9	male	neutered
(26)	57	Dominant	0.5	7	female	spayed
(27)	58	Dominant	0.625	8	female	spayed
(27)	59	Subordinate	-0.625	11	female	spayed
(28)	60	Dominant	0.25	4	male	intact
(28)	61	Subordinate	-0.5	12	female	spayed
(29)	62	Subordinate	-0.125	6	female	spayed
(29)	63	Dominant	0.125	9.5	female	spayed
(30)	64	Subordinate	-0.375	1	female	intact
(30)	65	Dominant	0.375	5	male	neutered
(31)	66	Subordinate	-0.125	11	female	spayed
(31)	67	Dominant	0.125	2.5	female	spayed
(32)	68		-0.25	9	male	neutered
(32)	69		-0.25	9.5	male	neutered
(32)	70	Dominant	0	11	male	neutered
