# Supplementary material for: High Rank, Low Tolerance: Hierarchy-Dependent Reactions of Cohabiting Companion Dogs to Being Separated from Their Owner
Source: Animals (Basel). 2026 Jun 25;16(13):1965. doi: 10.3390/ani16131965 (PMC13359868; doi:10.3390/ani16131965)
Supplement: Supplementary file 1 [file animals-16-01965-s001.zip › Table_Supplementary_2.pdf]

Supplementary Table S2. The structure of the five behavioral components, resulting from the Principal Component Analysis. In this study, we used the identical scoring system of the behaviors in the separation test as Lenkei et al. (2021) [55] did, thus, we relied on the component structure they described as well. The table shows the factor loadings for each coded behaviors. Factor loadings with bold indicate which principal component the behavior belongs to.

**Behavior**	**Chair**	**Escape**	**Whine–door**	**Bark–wagging**	**Sit**
Door distance	**−0.742**	0.159	0.123	−0.123	0.070
Chair exploration	**0.708**	0.048	0.174	−0.167	0.002
Chair distance	**0.694**	−0.243	−0.139	0.361	0.014
Chair orientation	**0.659**	0.141	0.091	−0.276	0.179
Rearing	−0.106	**0.832**	−0.093	0.115	0.053
Scratching	−0.204	**0.720**	0.115	0.134	0.081
Panting	0.304	**0.523**	−0.102	−0.220	−0.036
Moving	0.302	**0.494**	0.387	−0.219	−0.169
Laying	−0.052	−0.065	**−0.705**	−0.022	0.209
Whining	0.025	−0.145	**0.654**	0.287	0.246
Door exploration	−0.197	0.063	**0.635**	−0.189	−0.041
Barking/yelping	−0.033	0.470	−0.014	**0.610**	−0.059
Tail-wagging	0.203	0.334	0.220	**0.590**	−0.105
Exploration in general	0.086	0.059	0.289	**−0.528**	−0.186
Door orientation	−0.271	−0.108	0.274	**0.471**	0.064
Other vocalization	0.064	0.168	0.148	**0.349**	−0.031
Standing	−0.088	−0.195	0.285	0.084	**−0.833**
Sitting	−0.039	−0.109	0.242	0.049	**0.858**
**Cronbach's alpha**	0.7	0.64	0.57	0.52	0.69
**Variance explained**	13.2%	12.7%	10.8%	10.3%	9.3%
